# Notices, Etc., for April

**Published:** 1864-04

**Authors:** 


### NOTICES, ETC., FOR APRIL.

The American Tract Society, 28 Cornhill, Boston, and 13 Bible House, New-York, are with indefatigable industry issuing book after book as attractive in manner as in matter, so that all classes of readers may be supplied with spiritual and mental food. "Jerry and his Friends, or the Way to Heaven," by Alice A. Dodge, is full of interest and instruction. "Apples of Gold in Pictures of Silver," by Krune, contains eleven stories of practical use to all readers, old and young. Also "Letters to a Theological Student," by Leverett Griggs. Sargent's Temperance Tales, and a very valuable book of "Daily Prayers."

Agricultural Colleges are beginning to attract public attention. The great need of this country and its salvation from an oppressive national debt, is in intelligent farming; this will create gold in more incalculable quantities than the yield of the richest mines in California or Colorado. In this direction the Hon. Isaac Newton, with the assistance of his right-hand Secretary, James S. Grinnell, has given a sketch in the last bi-monthly report of the Agricultural Department for January and February, as to what studies these colleges should embrace, to wit, languages, mathematics, a geological museum, with a zoölogical department, maps, charts, philosophical instruments; a system of instruction for physical development, moral culture, drawing, land-surveying, book-keeping, normal school, model farm, military training, etc., etc. The report contains further a well-considered article on "The Future of American Cotton and Wool," tobacco cultivation, cattle-market, weights and measures, the weather, its effect on the farm, meteorological report, etc., etc. The Agricultural Department is not inferior in its importance on the future welfare of this country, to any other in the Government, and up to this time, it has been managed with an ability, wisdom, and judgment on the part of Messrs. Newton, Grinnell, with the aid of other gentlemen connected with this Bureau, which merits the thanks of this whole nation, and we trust our farmer readers, to whom the department sends its circulars for information and statistical statements on subjects connected with farming operations, will feel it to be a duty to themselves and to the country in general to be prompt, accurate, and painstaking, not only to give all the information they have within themselves, but to embody what they can collect from their neighbors, these very inquiries tending to excite a spirit of inquiry, investigation, and experiment, which will add millions to the national wealth eventually.

Vocal Gymnasium.—Prof. Hurlburt, of this city, gives instructions at the Cooper Institute, in private families and public schools, in the cultivation of the voice, and the proper development of the muscles of the chest and of respiration in general. How to read naturally and well, without fatigue or consciousness of effort, is a social accomplishment of more general use and practical employment than almost any other study in our schools, and we hope that the able and conscientious and indefatigable Professor will receive the patronage which he so well merits. To be able to read well is an accomplishment of which any one may be laudably proud.

### NOTICES.

"The Nation's Success and Gratitude." — Our old friend, David A. Sayre, the Kentucky banker, only a small part of whose princely benevolences were recorded in the August number of last year, has forwarded to us a discourse on the above subject, delivered on the last National thanksgiving-day by that stern old Presbyterian and loyal Unionist, Robert J. Breckenridge, the vigor of whose intellect has caused him for a quarter of a century past to stand a head and shoulders above the men of his time, and who in this last eloquent utterance shows that he is still as great in mind as he is as fearless in heart; and who will not join with him in his closing petition that a complete triumph and lasting peace may be speedily secured to us, by means which God will own and bless, and that he would incline and enable all men to walk in ways of wisdom, justice, and humanity?

Metropolitan Fair.—Surely it will cheer the hearts of our sick and wounded and imprisoned soldiers, as well as those who are now in the field, to learn that the wealth and the beauty and fashion of our great cities are making their very pleasures a means of enriching the treasury of the Sanitary Commission, a handsome contribution to which was realized on the evening of Saturday, March twelfth, on the occasion of a private concert, being the sixth of the series given at the princely mansion of one of our uptown millionaires, Dr. Thomas Ward, who, as on previous occasions, promptly and cordially gave up the use of the splendid music-room attached to his hundred-feet front, corner of Fifth Avenue and Forty-seventh street, to the committee of ladies who managed the whole affair. A more elegant company has perhaps never assembled on any similar occasion in this city. A long line of splendid equipages lined the avenue in the direction of the Central Park, while a double row reached up Forty-seventh street, (the handsomest and best built in New-York,) extending apparently to Sixth Avenue. The night and the weather were splendid, and every thing went off successfully, happily, and without a single occurrence to mar the enjoyment of the evening, which always is the case when Dr. Ward has the arrangement of affairs; not the least interesting feature of the occasion was the introduction of a part of the opera of the "Gipsy's Frolic," the words and music of which were composed by the Doctor himself, whose opinion, by the way, in all matters of taste, and music, and art is final in the circles of the upper-five. On the nineteenth, the seventh concert of the series takes place at the house of the Hon. August Belmont, in Fifth Avenue, which no doubt will do credit to the liberal banker.

W. J. Widdleton announces through the Publishers' Circular a new edition of "Health and Disease," to be ready without fail on the first of April. Orders from the trade solicited.

